# Colour change and colour phases in Lethrinidae with insights into ecology

**DOI:** 10.1002/ece3.10735

**Published:** 2023-12-06

**Authors:** Myriam E. Widmann, Sean van Elden, Jessica J. Meeuwig

**Affiliations:** ^1^ School of Biological Sciences The University of Western Australia Crawley WA Australia

**Keywords:** behaviour, camouflage, colour phase frequency, crypsis, stereo‐BRUVS

## Abstract

Colour change is used by a wide range of animals. It is used for intra‐ and interspecific communication and crypsis, and can occur on morphological and physiological levels. Bony fish employ rapid physiological colour change and display various types of patterns and colouration (colour phases) useful for aposematic and cryptic purposes. Using an existing database of benthic stereo‐baited remote underwater video systems from two locations in Western Australia, we tested whether the frequency of colour phases of emperors, Lethrinidae, varied by species. We described colour phases and rapid physiological colour change in 16 species of lethrinids, and related occurrences of colour change to feeding activity and life stages. Dark and light colour phases were observed in nine of the 16 evaluated species of which seven also displayed physiological colour change. Frequency of colour phases varied between species, suggesting that the display of different dark patterns may be especially important for certain species. Both juveniles and adults showed the ability to change between different colour patterns. The change into a mottled pattern mainly occurred while feeding or when approaching to feed, suggesting that it may be triggered by feeding and the associated decrease in environmental awareness. Colour change is a commonly observed strategy in lethrinids and may have evolved as an adaptation for increased foraging success or to reduce aggression from conspecifics. Physiological colour change allows lethrinids to quickly adapt to various cues from the environment and can therefore be considered a versatile physiological mechanism in this family.

## Graphical abstract

We described colour phases and rapid physiological colour change in 16 species of lethrinids, and related occurrences of colour change to feeding activity. Our results showed that frequency of dark phases varied between species, that both juvenile and adult lethrinids showed the ability to change between different colour patterns and that the most common pattern (the mottled pattern) was often observed while feeding. Physiological colour change is a commonly observed strategy in lethrinids and is most likely driven by different mechanisms, including increased foraging success and reduced aggression from conspecifics.

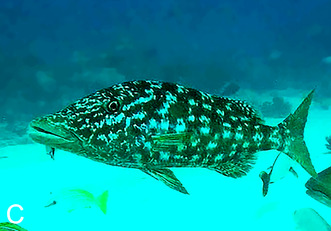

## 1 INTRODUCTION

Colour change is used by a wide range of animals, from mammals and reptiles to crustaceans, cephalopods and fish, and serves different purposes and strategies. Colour change is used for intra‐ and interspecific communication (Endler, 1978; Hemmi et al., 2006; Mäthger et al., 2009; Stoner et al., 2003; Sumner, 1911, 1940; Whiteley et al., 2009). Crypsis, also known as background matching, is a common purpose for colour change and used to both avoid and facilitate predation. Colour change includes several types of changes in physical appearance including colour, pattern, contrast, brightness and even 3D skin texture (Hanlon & Messenger, 2018). It can occur on both morphological (Leclercq et al., 2010; Sugimoto, 2002; Whiteley et al., 2009) and physiological levels (Ramachandran et al., 1996). Morphological colour change takes place over a period of days to months (Stuart‐Fox & Moussalli, 2009) while physiological colour change occurs rapidly (within seconds) (Ramachandran et al., 1996).

Rapid physiological colour change occurs through changes in pigment distribution of chromatophores, which are provided with many receptors and receive cues from external and internal sources (Fujii & Oshima, 1994). Melanophores, a type of chromatophore containing black and brown pigments (Fujii, 1969), normally play the most important role for changes in shades or hues and different colour patterns (Fujii, 1993, 2000). Mainly animals such as cephalopods, fish and amphibians show rapid physiological colour change that can be manipulated on an individual level and can be controlled and maintained by behaviour (Hanlon & Messenger, 1988; Ramachandran et al., 1996; Sumner, 1911, 1940). This type of colour change is beneficial when moving across spatially heterogeneous habitats and brings several advantages such as fast adaptation, adjustment and flexibility on an individual level in different environments and situations. Apart from crypsis (Sumner, 1911; Whiteley et al., 2009), this rapid colour change can be used for sexual display and communication (Kodric‐Brown, 1998; Sköld et al., 2008), as an anti‐predator strategy (Beeching, 1995; Endler, 1991; Fujii, 2000; Hanlon & Messenger, 1988 & Rodgers et al., 2010) or to increase foraging and predation success (Fujii, 2000). Moreover, it can be used to signal social status (Höglund et al., 2002) and dominance (Hamilton & Peterman, 1971), be a response to stress (Van der Salm et al., 2006; Wilson, 1998) or can serve as signal suppression (Beeching, 1995). Physiological colour change is therefore a crucial survival mechanism for various species.

Bony fish in particular exhibit physiological colour change and display various types of patterns and colouration useful for aposematic and cryptic purposes (Fujii, 1993, 2000). Physiological colour change in fish occurs by a synchronous movement of pigment granules in skin chromatophores or when light‐reflecting crystals of leucophores and iridophores, which are types of chromatophores containing colourless pigments, receive light from different angles (Fujii, 1969, 2000; Fujii & Oshima, 1994; Sumner, 1911). Different colour patterns are thought to be correlated with different behaviours and to be used for communication and signalling (Beeching, 1995). Various species of teleost fish have been found to use physiological colour change for functions such as background‐matching (Ramachandran et al., 1996), as a response to stress (Van der Salm et al., 2006) or predators (Cheney et al., 2008; Côté & Cheney, 2005; Tibblin et al., 2020), in the context of mutualistic interactions (Caves et al., 2018), aggression inhibition in conspecifics and anti‐predator tactic (Beeching, 1995) or mimicry (Cheney et al., 2008; Côté & Cheney, 2005). Perhaps the most well‐known example is the bluestriped fangblenny, *Plagiotremus rhinorhynchus*, *w*hich can facultatively mimic juvenile cleaner fish of the species *Labroides dimidiatus*, depending on whether this species is present (Cheney et al., 2008; Côté & Cheney, 2005). Aggressive mimicry is a useful strategy as fangblennies prey on body parts of other coral reef fish species and cleaner fish are allowed in close proximity to other fish (Côté & Cheney, 2005). Colour change in the three‐spined stickleback, *Gasterosteus aculeatus*, was found as a response to perceived predation risk, with higher expression when individuals were exposed to either olfactory cues or simulated attacks from a predator. These findings highlight the context dependence of colour change and how several factors may influence individual phenotypic flexibility (Tibblin et al., 2020).

Members of Lethrinidae, or emperors, are known to rapidly change between light and dark colour phases with varying patterns (Allen et al., 2003; Randall et al., 1998; Stuart‐Smith et al., 2015; Wilson, 1998). The family consists of five genera and 41 species (Froese & Pauly, 2000) and range in adult size up to 1 m in length (Kuiter, 2002). Most species primarily feed on sand‐dwelling invertebrates and can be found on the fringes of reefs. A few species also specialise on fish and are nocturnal predators. Rapid changes between pale and dark mottled patterns, spots, bars or stripes are documented particularly in the genus *Lethrinus* (Allen et al., 2003; Wilson, 1998). However, little is known about the frequency of different dark colour phases across species and the display of different colour patterns and physiological colour change.

We used an existing database of benthic stereo‐baited remote underwater video systems (stereo‐BRUVS) from two locations in north‐western Australia, Cocos Island and the Kimberley, to determine colour phases and physiological colour change in 16 species of lethrinids. Stereo‐BRUVS are a well‐established, non‐destructive sampling method for documenting abundance, diversity, biomass and size structure of marine communities (Cappo et al., 2006). Stereo‐BRUVS can be deployed across large spatial scales, to record large quantities of video data (Letessier et al., 2013) and to study behaviour in a range of animals as part of broader ecological studies, including fish (Fox & Bellwood, 2008), moray eels (Barley et al., 2016), cuttlefish (van Elden & Meeuwig, 2020) and sharks (Birt et al., 2019). The aims of this study were to test whether frequency of colour phases of lethrinids varied by species and determine rapid physiological colour change and the association with feeding behaviour. Additionally, different colour patterns were qualitatively described, and length of individuals were measured to distinguish between adults and juveniles. This analysis provides a first assessment with respect to the frequency of this colour change behaviour across a key predatory family in coral reef ecosystems.

## 2 MATERIALS AND METHODS

**FIGURE 1:**
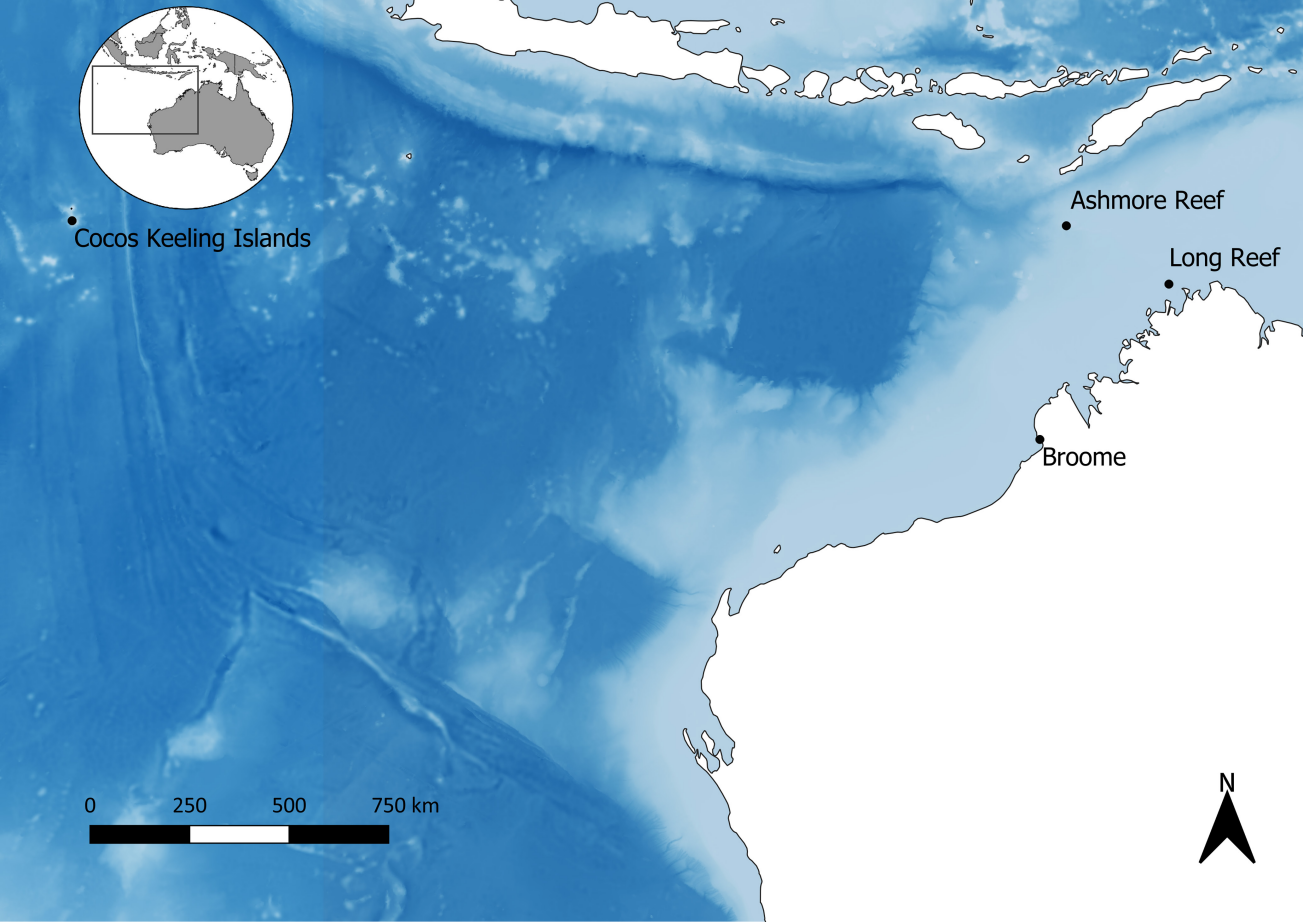
Position of two shallow marine coral reef sites in north‐western Australia surveyed in 2016 (The Cocos Keeling Islands) and 2017 (Kimberley composed of Long Reef and Ashmore Reef). The figure was created using ArcGIS 10.8.1.

Observations of lethrinids were extracted from an existing database of seabed stereo‐BRUVS footage built by the Marine Futures Lab at the University of Western Australia. The analysis focussed on two locations in north‐western Australia where lethrinids were commonly observed: the Cocos Keeling Islands, surveyed in 2016; and the Kimberley (comprised of Long Reef and Ashmore Reef), surveyed in 2017 (Figure 1). The abiotic habitat at both locations consisted of sand (grains <5 mm), rubble (grains >5 mm), low‐, medium‐ and high‐profile coral reefs. The biotic component consisted of macroalgae (turf, kelp, seaweed and sargassum), rhodoliths, sessile invertebrates and soft, patchy, branching, foliose, tabular, encrusting and massive corals.

Imagery was collected using standard methods for stereo‐BRUVS (Cappo et al., 2006; Langlois et al., 2018). BRUVS consisted of two waterproof high‐definition GoPro cameras with a convergent angle of 8°, mounted 80 cm apart on an aluminium rig with three legs, a bait arm of 1.8 m and a bait cage filled with ~1 kg of pilchards, *Sardinops sagax*. This bait is standardised across all seabed BRUVS deployments conducted by the Marine Futures Lab and was not selected to specifically attract lethrinids. Stereo cameras enable the observation and measurement of individuals passing through the visual field within about 10 m aided through attraction to the bait. The stereo‐BRUVS were deployed during daylight to exclude crepuscular behaviour (Myers et al., 2016), for 1 h per deployment at varying depths between 0.2 and 22.6 m. The videos were previously analysed by the staff of the Marine Futures Lab to determine diversity, abundance and size of all observed individuals using EventMeasure™ (SeaGIS Pty Ltd, 2017). A total of 61 BRUVS were available for the Cocos Islands and 161 for the Kimberley.

**FIGURE 2:**
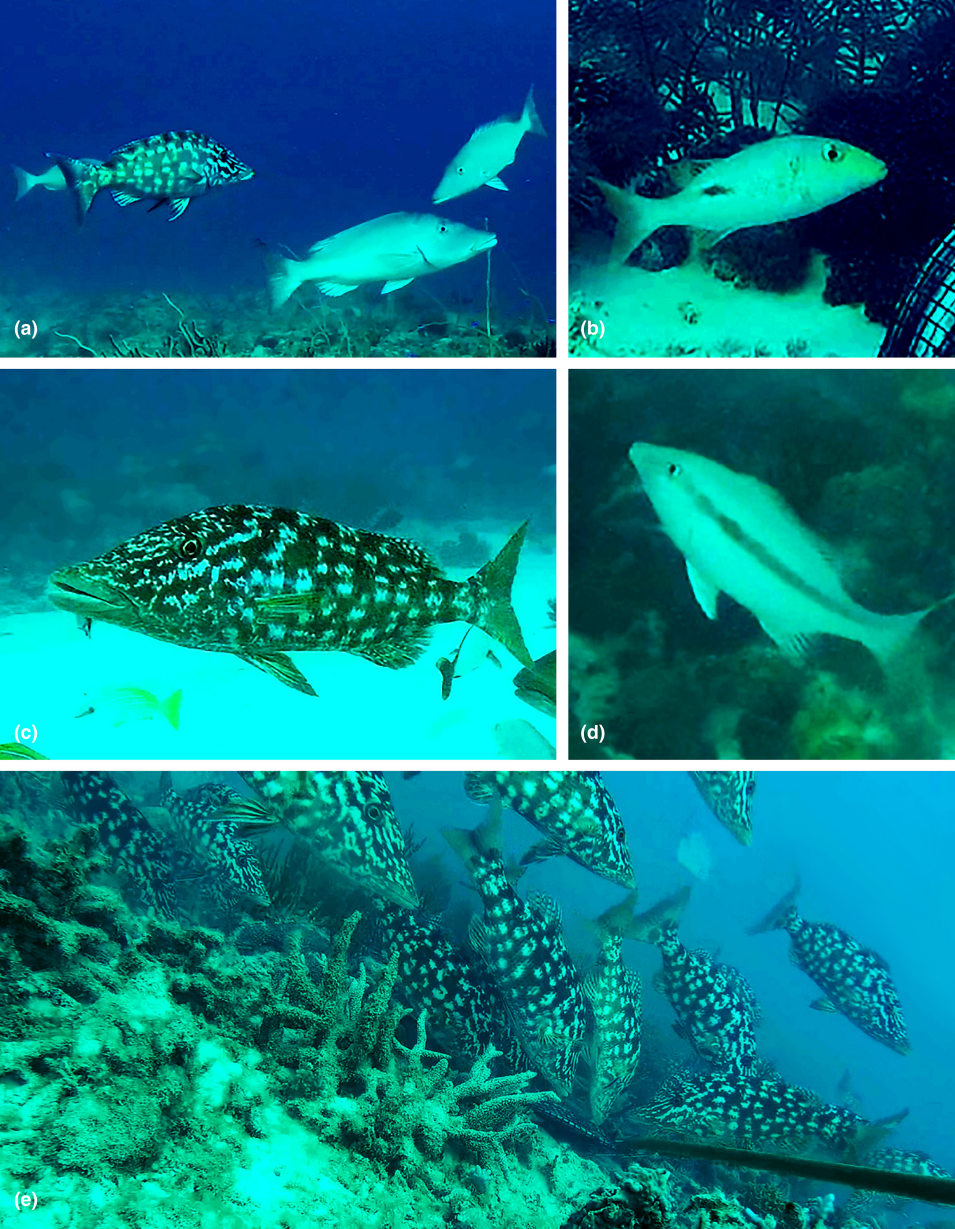
Light (a right) and dark (a left, b–e) colour phases of *Lethrinus olivaceus* (a, c–e) and *Lethrinus semicinctus* (b). Mottled pattern = a left, c and e (feeding behaviour can be observed as well), dark posterior middle blotch/dot = b, dark mid‐lateral stripe = d (juvenile). Some dark phases of *L. olivaceus* were feeding or approaching to feed (e). All shown individuals were adults except for one juvenile (d) and for the last picture, it was not clear whether juveniles were present (e).

All observations of individuals from the family Lethrinidae were extracted from the database. These observations were reanalysed using EventMeasure to determine frequency of different colour phases (number of individuals currently in each colour phase), physiological colour change (individuals actively displaying physiological colour change while in frame) and the association of physiological colour change with feeding behaviour (individuals displaying colour change while feeding or approaching to feed). Pictures taken from the videos were subsequently processed using Adobe Photoshop 22.2 (Adobe Inc., 2021) adjusting brightness, saturation and contrast. Distinction was made between two different colour phases, light (silver/pale) and any form of dark colour (partly or fully dark) (Figure 2). No quantitative differentiation of patterns among dark colour phases was made; however, different patterns were qualitatively described. Individuals displaying any form of dark colour phase were counted at the moment of MaxN (maximum number of individuals observed in the frame at one time) or the highest cumulative MaxN moment in which physiological colour change occurred or dark colour phases were present to avoid multiple counts of the same individuals. Individuals that showed active physiological colour change were also marked during dark phases.

Fork length (FL) of individuals was measured when possible, to distinguish between juveniles and adults. We distinguished between different life stages by comparing the measured length with maturity length (LM data on Fishbase) (Froese & Pauly, 2000). Conversion data from Fishbase were used to convert total length (TL) to FL when not given in FL to allow comparison. Length data of *Gymnocranius euanus* (sister taxon of *Monotaxis grandoculis*) from Fishbase were used to convert standard length (SL) to FL for *M. grandoculis* because no conversion data were available for this species.

Seven species in which dark and light phases were observed showed a greater observation number than 10, allowing quantitative analysis. For these species, a contingency chi‐square was performed to evaluate whether the frequency of dark and light colour phases varied between species.

## 3 RESULTS

### 3.1 Data and data quality

From the 61 BRUVS available for the Cocos Islands and 161 for the Kimberley, we recorded 559 individual lethrinids (Cocos Islands *n* = 150; The Kimberley *n* = 409), representing four genera and 16 species. The most commonly observed species was the longface emperor, *Lethrinus olivaceus* (*n* = 170), followed by humpnose big‐eye bream, *Monotaxis grandoculis* (*n* = 85), and orange‐striped emperor, *Lethrinus obsuletus* (*n* = 76) (Table 1). Only one individual of blue‐lined large‐eye bream, *Gymnocranius grandoculis*, three individuals of Ambon emperor, *Lethrinus amboninensis*, orange‐spotted emperor, *Lethrinus erythracanthus*, pink ear emperor, *Lethrinus lentjan*, and smalltooth emperor, *Lethrinus microdon*, were observed (Table 1).

**TABLE 1:** Number of individuals of 16 species of Lethrinidae showing dark and light colour phases, the proportion of dark phases and dark phases actively changing colour and the proportion of changes occurring while feeding.

Species		Colour phases			Dark phases		
Common name	Scientific name	Dark	Light	Total	Dark morphs (%)	Actively changing (%)	Changing while feeding (%)
Longface emperor	*Lethrinus olivaceus*	79	91	170	46.5%	63.3%	78.0%
Humpnose big‐eye bream	*Monotaxis grandoculis*	56	29	85	65.9%	7.1%	0.0%
Orange‐striped emperor	*Lethrinus obsuletus*	7	69	76	9.2%	28.6%	100.0%
Grass emperor	*Lethrinus laticaudis*	9	40	49	18.4%	66.7%	100.0%
Pacific yellowtail emperor	*Lethrinus atkinsoni*	4	31	35	11.4%	100.0%	75.0%
Longfin emperor	*Lethrinus erythropetrus*	0	31	31	0.0%	n/a	n/a
Spangled emperor	*Lethrinus nebulosus*	0	30	30	0.0%	n/a	n/a
Striped large‐eye bream	*Gnathodentex aureolineatus*	0	26	26	0.0%	n/a	n/a
Black blotch emperor	*Lethrinus semicinctus*	19	3	22	86.4%	21.1%	50.0%
Yellowlip emperor	*Lethrinus xanthochilus*	2	10	12	16.7%	50.0%	0.0%
Thumbprint emperor	*Lethrinus harak*	10	0	10	100.0%	n/a	n/a
Ambon emperor	*Lethrinus amboninensis*	0	3	3	0.0%	n/a	n/a
Orange‐spotted emperor	*Lethrinus erythracanthus*	0	3	3	0.0%	n/a	n/a
Pink ear emperor	*Lethrinus lentjan*	1	2	3	33.3%	0.0%	n/a
Smalltooth emperor	*Lethrinus microdon*	2	1	3	66.7%	0.0%	n/a
Blue‐lined large‐eye bream	*Gymnocranius grandoculis*	0	1	1	0.0%	n/a	n/a
Total		189	370	559	33.8%	37.6%	73.2%

*Note*: Not applicable (n/a) refers to where no individuals showed dark phases or where no feeding individuals were observed as distinct from 0% which represents that none of the found phases were dark, none of the dark phases were actively colour changing, or none of the actively changing individuals were observed to feed (*n* = 559).

### 3.2 Occurrence and frequency of colour phases and colour change

**FIGURE 3:**
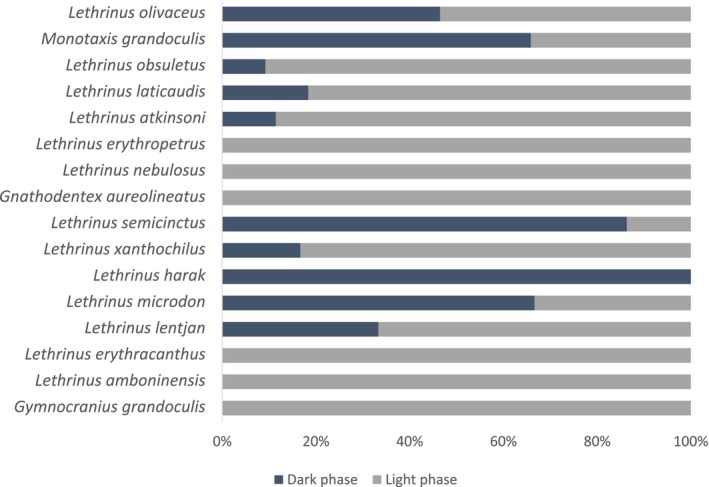
Percentage of individuals of 16 species of lethrinids showing dark and light colour phases: *Lethrinus olivaceus* (*n* = 170), *Monotaxis grandoculis* (*n* = 85), *Lethrinus obsuletus* (*n* = 76), *Lethrinus laticaudis* (*n* = 49), *Lethrinus atkinsoni* (*n* = 35), *Lethrinus erythropetrus* (*n* = 31), Lethrinus nebulosus (*n* = 30), *Gnathodentex aureolineatus* (*n* = 26), *Lethrinus semicinctus* (*n* = 23), *Lethrinus xanthochilus* (*n* = 12), Lethrinus harak (*n* = 10), Lethrinus *amboninensis* (*n* = 3), *Lethrinus erythracanthus* (*n* = 3), *Lethrinus lentjan* (*n* = 3), *Lethrinus microdon* (*n* = 3) and *Gymnocranius grandoculis* (*n* = 1).

Light and dark colour phases were observed in nine of the 16 species, only light colour phases were observed in six species and only dark colour phases were observed in one species (Table 1; Figure 3).

A minimum of 10 total observations were found in 11 of the 16 species, including seven of which displayed dark and light colour phases. For the remaining species, observed numbers were too low to reliably estimate the frequency of colour phases. The highest percentage of individuals observed in their dark colour phase was found in *L. semicinctus* followed by *M. grandoculis* and *L. olivaceus* (Table 1, Figure 3.). Rapid physiological colour change (actively changing while in frame) was observed in 37.6% (*SD* = 34.8, *Min* = 7.1, *Max* = 100) of the cases where a dark phase was recorded and was observed in *L. olivaceus*, *M. grandoculis*, *L. obsuletus*, *L. laticaudis*, *L. atkinsoni*, *L. semicinctus* and *L. xanthochilus* (Table 1). In all other instances, individuals were already in their dark phase when entering the frame and did not display physiological colour change while still in the frame. For the seven species that actively changed colour, an average of 73.2% (*SD* = 42.8) did so while feeding. Active colour change while feeding varied by species; *M. grandoculis* and *L. xanthochilus* did not exhibit active physiological colour change when feeding, while *L. obsuletus* and *L. laticaudis* actively changed colour when feeding 100% of the time (Table 1). The frequency of dark phases varied significantly by species (χ^2^_[6, *n* = 449]_ = 101.2, *p* < .001).

Length measurements were possible in six of the species that displayed light and dark phases, allowing a distinction between life stages and each colour phase. In adult individuals of *L. atkinsoni*, light and dark phases were observed, whereas in juveniles of this species only light phases were found (Table 2). The remaining species displayed only light or dark phases in each life stage or no distinction between life stages (*L. amboinensis*) was possible due to a lack of length measurement data.

**TABLE 2:** Number of individuals (*N*) and proportion of individuals (*%*) showing dark and light colour phases of six species of Lethrinidae separately for each life stage (adults and juveniles) (*n* = 236).

	Species		Dark phase		Light phase		Total
	Common name	Scientific name	*N*	%	*N*	%	*N*
Adults	Black blotch emperor	*Lethrinus semicinctus*	5	83.33	1	16.67	6
	Grass emperor	*Lethrinus laticaudis*	4	33.33	8	66.67	12
	Humpnose big‐eye bream	*Monotaxis grandoculis*	5	71.43	2	28.57	7
	Longface emperor	*Lethrinus olivaceus*	27	45.00	33	55.00	60
	Orange‐striped emperor	*Lethrinus obsuletus*	1	5.00	19	95.00	20
	Pacific yellowtail emperor	*Lethrinus atkinsoni*	3	37.50	5	62.50	8
	Total		45	40	68	60	113
Juveniles	Black blotch emperor	*Lethrinus semicinctus*	4	50.00	4	50.00	8
	Grass emperor	*Lethrinus laticaudis*	2	22.22	7	77.78	9
	Humpnose big‐eye bream	*Monotaxis grandoculis*	41	80.39	10	19.61	51
	Longface emperor	*Lethrinus olivaceus*	7	26.92	19	73.08	26
	Orange‐striped emperor	*Lethrinus obsuletus*	2	10.00	18	90.00	20
	Pacific yellowtail emperor	*Lethrinus atkinsoni*	0	0.00	9	100.00	9
	Total		56	45.53	67	54.47	123

### 3.3 Qualitative colour pattern identification

**FIGURE 4:**
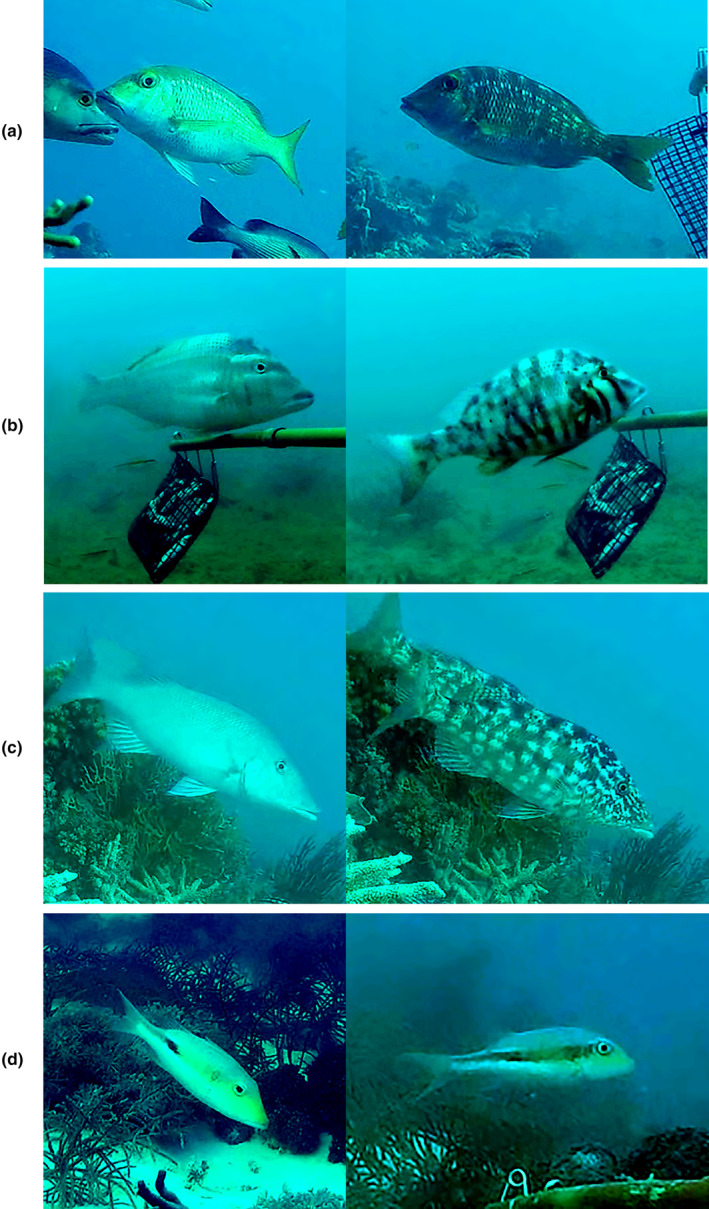
Adults of *Lethrinus atkinsoni* (a), *L. laticaudis* (b), *L. olivaceus* (c) and *L. semicinctus* (d) before (left) and after (right) colour change. Light colour phases = a, b and c left, mottled pattern = a, b and c right, dark posterior middle blotch/dot = d left, dark lateral stripe = d right.

A mottled pattern was observed in adults and juveniles of *L. semicinctus*, *L. laticaudis* and *L. olivaceus*, in adults of *L. obsuletus* and *L. atkinsoni* and in the observed juvenile (*n* = 1) of *L. xanthochilus*. The display of the mottled pattern was observed in 90.4% (*n* = 47) of the times colour change was observed in direct relation to feeding (feeding or approaching to feed) (Figure 2e). In the remaining 9.6% (*n* = 5) of colour change in association with feeding, individuals either displayed a dark lateral stripe or dot, or changed between those two and the mottled pattern. The dark mid‐lateral (horizontal) stripe was observed in adults and juveniles of *L. olivaceus* and *L. microdon* (only one observation for adults and juveniles in *L. microdon*), in adults of *L. semicinctus* and in the one juvenile observed of *L. lentjan*. Dark phases of juveniles and adults of *M. grandoculis* were either partly (dorsal area) or completely dark and no mottled pattern was observed. Rapid physiological colour change and the display of these patterns was observed in adults of *L. obsuletus* and *L. atkinsoni* (Figure 4a) adults and juveniles of *L. laticaudis*, *L. olivaceus*, *L. semicinctus* (Figure 4b–d) and *M. grandoculis*, and in the juvenile of *L. xanthochilus*. In *Lethrinus semicinctus*, a dark posterior middle dot in addition to the mottled pattern, in adults and juveniles and a horizontal stripe in adults was observed. Furthermore, the middle dot occasionally faded away, leaving the fish completely pale or changed into the dark mid‐lateral stripe (Figure 4d). All observed individuals (juveniles and adults) of *L. harak* also showed a dark dot/spot; however, no change into a complete light (pale) form or a mottled pattern was observed.

## 4 DISCUSSION

Physiological colour change occurs in a range of lethrinid species and was observed in both adults and juveniles. Colour change was shown to be frequent in some species of Lethrinidae and the frequency of dark colour phases varied significantly between species. This variation between species could be explained by the influence of heritable genetic components (Bell et al., 2009; Boake, 1989; Tibblin et al., 2020) and suggests that the display of dark patterns may be especially important for certain species. Although physiological colour change was not seen in adults and juveniles of all species, the results suggest that colour change is not limited to either juveniles or adults. Furthermore, we observed some patterns in adults that were previously described as typical for juveniles (Wilson, 1998). It remains unclear whether these patterns are equally used by juveniles and adults or are used by adults to mimic juveniles. As described in other fish species, mimicking juveniles can lead to decreased or inhibited aggression (Beeching, 1995) or could be used to increase predation success (Cheney et al., 2008; Côté & Cheney, 2005). However, the purpose of adult individuals mimicking typical juvenile patterns remains unclear in lethrinids.

Rapid physiological colour change and the associated occurrence of dark phases could still occur in species in which dark phases and/or colour change was not observed. The lack of observed physiological colour change in some species can be explained by the rather small sample size for these species. Although, *L. erythracanthus* is characterised by morphological colour change throughout its life history, no physiological colour change and a lack of dark pattern have been previously described for this species (Wilson, 1998). This is confirmed by our results. However, it needs to be interpreted with caution as we only observed three individuals. Colour change into dark phases in *L. harak* has previously been described as rapid intensification of a dark mid‐body spot (Allen et al., 2003) and in juveniles the display of a mid‐lateral stripe or, if stressed, the appearance of dark bands *L. harak* (Wilson, 1998). Colour change in juveniles of *L. lentjan* and *L. nebulosu*s were also observed previously (Wilson, 1998). This suggests that at least these species are able to change between dark and light colour phases, although it was not observed in this study.

A dark mid‐lateral stripe, displayed in undisturbed situations or while foraging, is known as a typical pattern for juveniles of some species of lethrinids (Carpenter & AlIen, 1989; Wilson, 1998). One possibility is that this pattern could be used for aposematic purposes and to confuse piscivores visually (predator avoidance) as it can be associated with poisonous juvenile siganids (Wilson, 1998). Here, the mid‐lateral stripe was also observed in adults of *L. semicinctus*, *L. olivaceus*, *L. obsuletus* and *L. microdon*, suggesting it is either used by adults for the same reasons as in juveniles or to mimic juveniles. Primary stripes, another pattern used as a stress or fright response (Wilson, 1998) and to probably signal danger (Rowland, 1979; Wilson, 1998) were described in early juveniles of *L. lentjan* and *L. obsuletus* (Wilson, 1998). This pattern, however, was not found in the current study, suggesting that other patterns may have replaced this colouration. Other patterns could be more beneficial when these animals increase in size.

The mottled pattern was the most commonly observed dark pattern and occurred in adults and juveniles. In juveniles, this pattern is described as a response to any kind of disturbance and stress situation, to be used in antagonistic interactions with conspecifics (Wilson, 1998), or to function as an alarm signal or visual warning (Hamilton & Peterman, 1971). Disruptive colouration as found in the mottled pattern is suggested to provide an increased level of camouflage compared to background matching (Merilaita, 1998) because it breaks up the animal's body outline by increasing the contrast of the pattern (Hanlon & Messenger, 1988; Kelman et al., 2007; Schaefer & Stobbe, 2006). It therefore may be the most beneficial pattern used to decrease detectability in the surrounding environment. The mottled pattern was observed in six out of seven species in which colour change occurred and was also the most common pattern associated with feeding behaviour. This suggests that the mottled pattern may be associated with feeding behaviour; however, this still needs to be quantitatively evaluated and compared to other behaviours and stimuli.

Rapid physiological colour change was mainly observed while feeding or when approaching to feed, which indicates that this type of colour change may be triggered by feeding. In *M. grandoculis* and *L. xanthochilus*, no change while feeding was observed suggesting that colour change may not be associated with feeding in these species. However, the sample size of dark phases was very small for *L. xanthochilus* and no reliable conclusion could be drawn whether feeding is associated with physiological colour change in this species. Dark colour patterns in fish are often used for aposematic or cryptic purposes (Fujii, 1993, 2000; Sumner, 1911; Whiteley et al., 2009), which could explain the high percentage of changing into dark phases while feeding as the animals are less able to pay attention to predators and potentially use certain patterns to decrease the risk of being detected. Colour change while approaching to feed could also be a reaction to background objects and environmental cues (Barbosa et al., 2008; Hanlon et al., 2009). A comparison with other behaviours and visual cues of the environment is needed to evaluate feeding as a trigger for physiological colour change in lethrinids. It was often observed that when a large group of fish already had changed, all other incoming individuals were changing into dark phases. This could be explained by intraspecific communication to signal feeding or social status (Höglund et al., 2002), serve as an aggression inhibitor strategy (Beeching, 1995) or shoaling strategy (Rodgers et al., 2010; Wilson, 1998).

While we did not have sufficient measurements to allow for statistical analyses, we observed a general trend of lethrinids measured in dark phase being smaller than those in light phase. Juveniles are known for colour change and the increased use of dark patterns due to their especially high predation risk (Wilson, 1998). Smaller adults may have exhibited the same use of colour change as juveniles based on their size or because mimicking juveniles could also be especially rewarding for small sized adults. However, a larger sample size of measured individuals would be required to evaluate whether the use of colour change is driven by body size.

## 5 CONCLUSIONS

Rapid colour change and the related occurrence of light and dark colour phases is a frequently observed strategy in lethrinids and was found to be present in adults and juveniles. The frequency of dark colour phases varied between species, suggesting that the display of dark patterns may be especially important for certain species and may be influenced by heritable genetic components. The most common pattern associated with colour change was the mottled pattern. This pattern is likely to decrease detectability in the surrounding environment and may be the most beneficial pattern used for camouflage and cryptic purposes. Colour change into the mottled pattern was often observed while feeding, suggesting it may be triggered by feeding and the associated decrease in environmental awareness. Remote video sampling techniques such as stereo‐BRUVS can capture novel and rare behaviours such as colour change and help us understand how species interact with their environment and with other marine life. We found that rapid physiological colour change can be commonly observed in lethrinids, especially in the context of feeding. It is therefore most likely used to increase their foraging success and possibly to reduce aggression from conspecifics. Colour change in lethrinids can therefore be considered a versatile physiological mechanism which allows these species to quickly adapt to various cues from their environment.

## AUTHOR CONTRIBUTIONS

**Myriam E. Widmann:** Conceptualization (lead); data curation (lead); formal analysis (lead); investigation (lead); writing – original draft (lead); writing – review and editing (equal). **Sean van Elden:** Conceptualization (supporting); data curation (supporting); formal analysis (supporting); investigation (supporting); writing – original draft (supporting); writing – review and editing (equal). **Jessica J. Meeuwig:** Conceptualization (supporting); data curation (supporting); formal analysis (supporting); investigation (supporting); writing – original draft (supporting); writing – review and editing (equal).

## CONFLICT OF INTEREST STATEMENT

The authors declare no competing interests.

## Data Availability

All survey data are publicly accessible via Dryad (https://datadryad.org/stash/share/UokX4GwkSePTQ4nNrWEsJoei25NNqLKv3tCayg4n1Jc).
